# Blood pressure levels and body mass index in Brazilian adults with Down syndrome: Níveis de pressão arterial e índice de massa corporal em adultos brasileiros com síndrome de Down

**DOI:** 10.1590/1516-3180.2016.0057180316

**Published:** 2015-11-13

**Authors:** Felipe Pucci, Guilherme Machado, Edcarlo Solera, Fernanda Cenovicz, Christian Arruda, Chiu Braga, Renato Nisihara

**Affiliations:** 1 MD. Attending Physician, Department of Medicine, Universidade Positivo (UP), Curitiba, PR, Brazil.; 2 Undergraduate Student, Department of Medicine, Universidade Positivo (UP), Curitiba, PR, Brazil.; 3 PhD, Assistant Professor, Department of Medicine, Universidade Positivo (UP), Curitiba, PR, Brazil.

**Keywords:** Down syndrome, Blood pressure, Obesity, Body mass index, Adult, Síndrome de Down, Pressão sanguínea, Obesidade, Índice de massa corporal, Adulto

## Abstract

**CONTEXT AND OBJECTIVE:**

Increased life expectancy among people with Down syndrome (DS) has introduced new environmental factors that may affect blood pressure (BP) and/or lead to obesity in this population. The aim here was to investigate BP levels and body mass index (BMI) in adults with DS, correlating these data with the patients' sex and age.

**DESIGN AND SETTING:**

Analytical cross-sectional observational study conducted in special schools in Curitiba (PR), Brazil.

**METHODS:**

97 adult patients were included. BP was measured in accordance with the established guidelines. BMI was calculated by dividing the weight by the height squared (kg/m^2)^.

**RESULTS:**

Sex had no influence on BMI; nor did systolic BP (SBP) or diastolic BP (DBP). The age range was from 18 to 56 years. No correlation was observed between increasing age and greater BMI or BP. Eighty-six individuals (88.7%) presented normal BP, eleven (11.3%) prehypertension and none hypertension. Twenty patients (20.4%) presented BP lower than 90 × 60 mmHg. BMI ranged from 18 to 48 kg/m^2^ (mean of 28.8 ± 3.92 kg/m^2)^: 21.9% had normal weight; 40.7% were overweight; and 25.3% had obesity class I, 9.9% class II and 2.2% class III. Higher BMI was associated with significantly greater SBP and DBP (P = 0.0175 and P = 0.0015).

**CONCLUSION:**

Sex and age did not influence SBP, DBP or BMI in Brazilian adults with DS. Higher BMI was associated with greater BP (both systolic and diastolic).

## RESUMO

**CONTEXTO E OBJETIVO:**

O aumento da expectativa de vida das pessoas com síndrome de Down (SD) introduziu novos fatores ambientais que podem afetar a pressão sanguínea e/ou levar a obesidade nessa população. O objetivo foi investigar os níveis de pressão arterial (PA) e o índice de massa corporal (IMC) em adultos com SD, correlacionando estes dados com a idade e o gênero dos pacientes.

**DESENHO E LOCAL:**

Estudo observacional, transversal e analítico, realizado em escolas especiais em Curitiba (PR), Brasil.

**MÉTODOS:**

Foram incluídos 97 pacientes adultos. A aferição da PA foi feita de acordo com as diretrizes estabelecidas. O IMC foi calculado dividindo-se o peso pela altura ao quadrado (kg/m^2)^.

**RESULTADOS:**

O gênero não influenciou o IMC, a pressão arterial sistólica (PAS) e diastólica (PAD). A idade variou de 18 a 56 anos. Não foi observada correlação entre aumento da idade e maior IMC ou pressão arterial. 86 indivíduos (88,7%) apresentaram PA normal, 11 (11,3%) pré-hipertensão e nenhum hipertensão. Vinte (20,4%) apresentaram PA inferior a 90 × 60 mmHg. O IMC variou entre 18 e 48 kg/m^2^ (média de 28,8 ± 3,92), 21,9% tinham peso normal, 40,7% sobrepeso, 25,3% obesidade grau I, 9,9 % grau II e 2,2% obesidade grau III. Aumento significativo da PAS e PAD foi associado com elevação do IMC (P = 0,0175 e P = 0,0015).

**CONCLUSÃO:**

Verificou-se que sexo e idade não influenciaram PAS, PAD e IMC em adultos brasileiros com SD. O aumento significativo da PAS e PAD foi associado com o aumento do IMC.

## INTRODUCTION

Over the last five decades, there has been a trend toward longer survival among individuals with Down syndrome (DS).^1^^,^^2^^,^^3^ In developed countries, recent estimates have indicated that their average age at death is greater than 50 years.^2^^,^^3^ Reduced institutionalization with increased mobility and integration into society has also played a role.^1^^,^^4^^,^^5^

DS is a multiorgan disorder, affecting the heart and vascular system both structurally and functionally.^6^ Conditions such as obesity, mobility restrictions, depression, hypothyroidism and Alzheimer's disease are known to become increasingly prevalent in later life,^7^ including increased blood pressure (BP) and body mass index (BMI). There are a few studies evaluating blood pressure in adults with DS that have reported that their BP is lower than that of the general population and people with other forms of mental handicap. However, some of these studies were conducted many years ago.^8^^,^^9^ Today, individuals with DS are exposed to different environmental factors such as stress, fast foods, greater social inclusion and new challenges that can affect BP and cause obesity.

Draheim et al.^10^ investigated 52 DS patients and reported that systolic and diastolic BP were signiﬁcantly lower in adults with DS than in a matched control group; and that their body mass index (BMI) was 31.0 ± 6, which was significantly higher than that of the controls (28.0 ± 7). There are no studies on this topic conducted in Brazil.

## OBJECTIVE

In the present study, we investigated the levels of diastolic/systolic BP and BMI among noninstitutionalized adults with DS in Curitiba, Brazil, correlating the findings with the patients' sex and age.

## METHODS

This study had an observational cross-sectional design and was approved by the local research ethics committee (Positivo University, number 297.349/2013). The parents or guardians of all participants signed informed consent forms.

The study population comprised adults with DS over the age of 18 years who were being followed up at two institutions: Associação de Pais e Amigos dos Excepcionais (APAE-PR) and Associação Reviver, both in Curitiba, Paraná. The parents were sent a letter proposing the study. If they agreed, they signed the consent form and the patient was included in the study. In a brief questionnaire, the participants answered questions about the presence of diagnosed hypertension, smoking and alcohol consumption. The participants received guidance on healthy habits and were encouraged to practice physical activity. The study was conducted over the period from May 2013 to September 2013.

The researchers invited 97 consecutive adults with DS who were attending the schools that collaborated in the study. All agreed in to participate. Thus, a total of 97 DS patients (49 male and 48 female patients) in Curitiba, Paraná, Brazil, were included in the study. None of them were smokers or alcohol consumers or had hypertension diagnosed previously.

BP measurements were made using the auscultatory method with a calibrated mercury sphygmomanometer, performed by an operator trained in the standardized technique. The patients were properly prepared and positioned. They remained seated quietly for at least five minutes on a chair, with feet on the floor and arms supported on armrests at heart level. Caffeine and exercise needed to be avoided for at least 30 minutes prior to the measurements, in accordance with the guidelines established by the American Heart Association in the Seventh Report of the Joint National Committee (JNC-7).^11^ The measurements made by the two researchers agreed well when simultaneous recordings were made in the right and left arms of the patients, with appropriate cuff sizes used. For all the patients in this study, three BP measurements were made, done on different days within one week. The mean from these measurements was used in the analyses.

BMI was calculated by dividing the weight in kilograms by the height in meters squared (kg/m^2)^, in accordance with the World Health Organization (WHO) recommendations.^12^ To obtain the patients' body weight and height, we used an electric scale and a tape measure. Patients who presented abnormal BP measurements received a letter from the school, in which their parents were advised to seek the municipal health service for the patients' BP to be measured. The healthcare professional assigned to the special school was also informed about the findings. All the patients received information about proper nutrition and physical exercise for maintaining their health.

The data distribution was analyzed using the Kolmogorov-Smirnov test. The results were expressed as the mean and standard deviation (SD) for parametric data and as the median and interquartile range (IQR) for nonparametric data. The Fisher exact and chi-square tests were used for association studies on nominal data and the unpaired t test and Mann-Whitney test were used for continuous data. Calculations were done with the aid of the GraphPad Prism software, version 4.0, and Medcalc version 12.1.3.0. The significance level used was 5%.

## RESULTS

Among the 97 DS patients, 49 (51%) were male. There were no statistically significant differences according to sex with regard to evaluations on BMI (P = 0.84), systolic blood pressure (SBP) (P = 0.64) or diastolic blood pressure (DBP) (P = 0.33), as demonstrated in Table 1.

**Table 1:**
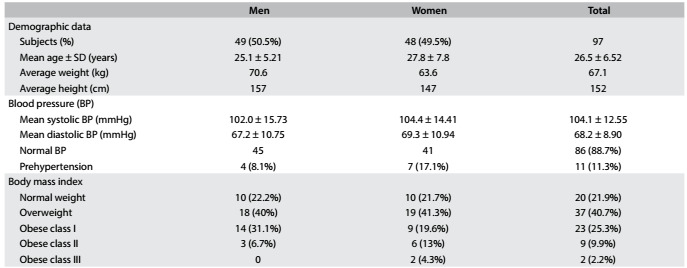
Clinical and demographic data and comparison between sexes in the study group

SD = standard deviation.

The population age range was from 18 to 56 years, with a mean age of 26.5 ± 6.52 years; 74.2% were less than or equal to 30 years of age (Table 1). The statistical analysis showed that there was no association between increasing age and higher BMI (P = 0.64), or between increasing age and SBP (P = 0.45) or DBP (P = 0.80).

The systemic arterial pressure ratios of the 97 patients evaluated in this study ranged from 72/36 mmHg to 132/88 mmHg. Eighty-six of them (88.7%) presented BP below the "optimal" reference value (120/80 mmHg) for individuals with normal BP, as recommended through JNC-7.^11^ Eleven patients (11.3%) were classified as presenting prehypertension and no patient presented hypertension (Table 1). Three patients (3.1%) had borderline BP, defined as SBP 130-139 mmHg and another two patients (2.1%) showed DBP between 85 mmHg and 89 mmHg. On the other hand, twenty patients (20.4%) presented BP levels lower than 90 mmHg × 60 mmHg (10 males; mean age of 26.4 years): seven individuals with normal BMI, 10 with obesity class I and three with obesity class II. Four of them had systemic BP of 70 mmHg × 50 mm/Hg.

BMI screening was performed on 91 patients. Six patients did not allow weight and height measurements to be made. The BMI ranged from 18 to 48 kg/m^2^, with a mean of 28.8 kg/m^2^ ± 3.92 kg/m^2^. Among the patients, 21.9% presented weight within normal limits, while 40.7% were classified as overweight, 25.3% as obese class I, 9.9% as obese class II and 2.2% as obese class III, in accordance with the World Health Organization classification system^12^ (Table 1).

**Figure 1:**
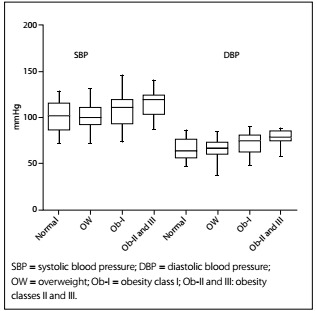
Blood pressure levels in patients with Down syndrome, according to body mass index.

Higher BMI was associated with significant greater SBP and DBP (P = 0.0175 and P = 0.0015, respectively). Figure 1 shows the SBP and DBP values according to BMI variation. No significant correlation was observed when the patients' ages were compared with either SBP or DBP. Among the patients with normal weight (21.9%), the median BP was 101 mmHg x 65 mmHg. The overweight patients (40.7%) had median BP of 102 mmHg x 66 mmHg and those with obesity class I (25.3%) had median BP of 105 mmHg x 70 mmHg. BP was significantly greater among the patients with obesity classes II and III (12.1%), who showed median BP of 115 mmHg x 80 mmHg (P = 0.0038), in comparison with the patients with normal weight.

## DISCUSSION

In this study, we evaluated the BP and the BMI of adults with DS. We showed that the diastolic and systolic BPs were lower among individuals with DS and that high BMI was associated with increased BP.

In terms of BP, in our study, the majority (88.7%) of the patients had BP below the recommended levels, which is consistent with the findings of other authors.^8^^,^^10^ Among our patients, none presented hypertension; 11 cases (11.3%) were classified as presenting prehypertension, among which 9 presented obesity class II and III. These patients will be monitored through periodic measurements of their BP and referral for medical care. On the other hand, 20.6% of the patients presented low systemic blood pressure, i.e. below 90 mmHg x 60 mmHg, without any association with sex or age. Some authors have suggested that this low BP may be associated with Alzheimer's disease and premature aging among individuals with DS.^8^ On the other hand, the lack of influence of sex and age on BP and BMI may be due to the small sample size or to survival bias, in the case of age.

A sedentary lifestyle and an unhealthy diet are common among individuals with DS, especially among those living in community settings.^13^^,^^14^ The prevalence of overweight and obesity among patients with DS is significantly higher than among other patients with intellectual disabilities or in the general population. The reported prevalence of obesity among adults with DS is between 31% and 47%.^15^^,^^16^^,^^17^ In our study, the prevalence of obesity among adults with DS was 37.8%, and 40% of the population was overweight. Only 22.2% presented appropriate weight for age. It is natural to emphasize the importance of consistent exercise, good diet, community involvement and regular health examinations for these individuals. In a study on physical inactivity among adults with intellectual disability, Draheim et al*.* ^18^ reported that less than 46% of the men and women participated in the recommended amount of physical activity and that no adults older than 30 years reported participation in vigorous physical activity. However, Fujiura et al.^19^ founded that there were no strong links between BMI, diet and exercise among adults with DS. They discovered a significant link between friendships or access to recreation and BMI, concluding that community interactions had a major effect on health. In our view, healthy behavior should be stimulated. It is to be expected that a specific approach is needed to get these people interested and motivated to change their lifestyle. Therefore, development of specific programs for DS patients that may be conducted in schools is necessary.

## CONCLUSION

In our study, we found that sex and age did not influence SBP, DBP or BMI among Brazilian adults with DS. We observed that our DS patients presented high BMI. Furthermore, higher BMI was associated with higher systolic and diastolic BP.
